# Supplementary material for: Carbon for nutrient exchange between Lycopodiella inundata and Mucoromycotina fine root endophytes is unresponsive to high atmospheric CO2
Source: Mycorrhiza. 2021 Apr 21;31(4):431–40. doi: 10.1007/s00572-021-01033-6 (PMC8266774; doi:10.1007/s00572-021-01033-6)
Supplement: Supplementary file 1 — Supplementary file1 (DOCX 505 KB) [file 572_2021_1033_MOESM1_ESM.docx]

**Supplementary information**

**Mycorrhiza – Original Paper**

**Carbon for nutrient exchange between *Lycopodiella inundata* and Mucoromycotina fine root endophytes is unresponsive to high atmospheric CO_2_.**

Grace A. Hoysted^1*^, Jill Kowal^2^, Silvia Pressel^3^, Jeffrey G. Duckett^3^, Martin I. Bidartondo^2,4^, Katie J. Field^1^

**This file contains the following information:**

**Figure S1:** Schematic diagram and photograph of radio and stable isotope tracing system for a lycophyte host plant.

**Figure S2:** Comparison of colonisation of root segments colonised by CO_2_ treatment.


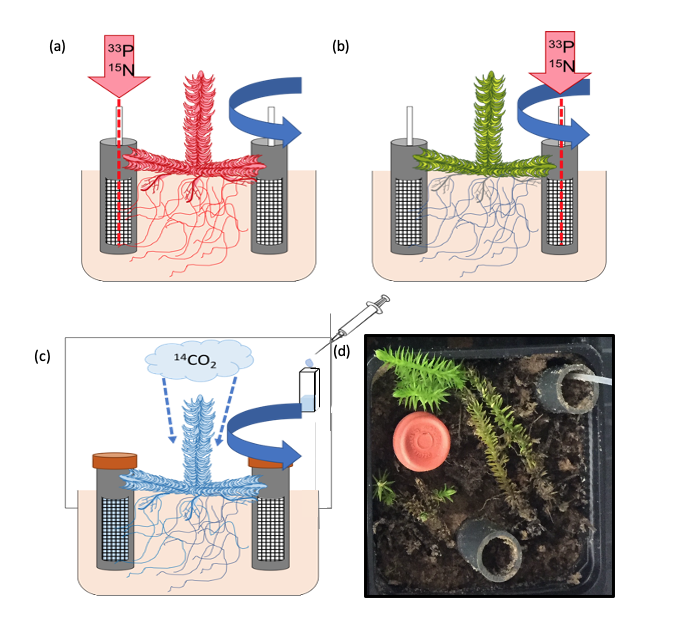


**Figure S1**. Schematic diagram and photograph of radio and stable isotope tracing experiment for a lycophyte host plant. (a) Microcosm containing static core in which ^33^P and ^15^N was injected; (b) Microcosm containing rotated core in which ^33^P and ^15^N was injected; (c) Microcosm set-up enclosed in an air-tight container in which ^14^C isotope tracing was conducted and (d) Photograph of *Lycopodiella inundata*-MFRE radio- and stable isotope tracing experimental system.

a)


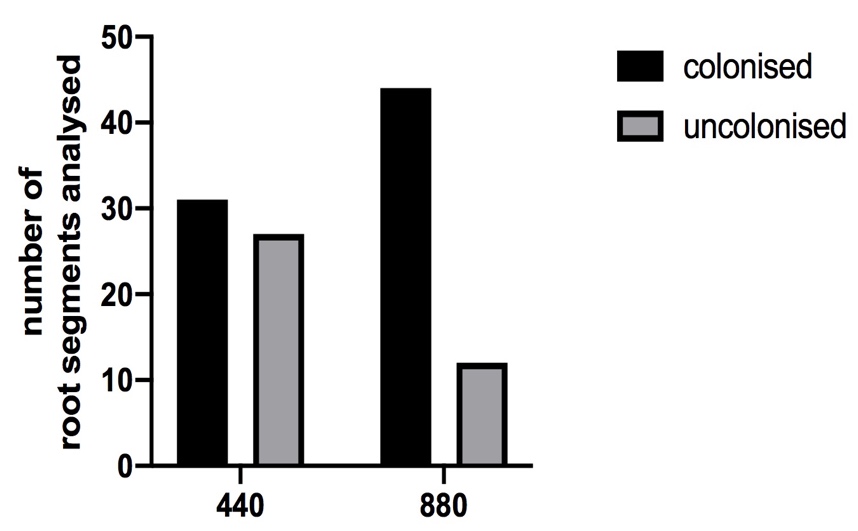


**

b)


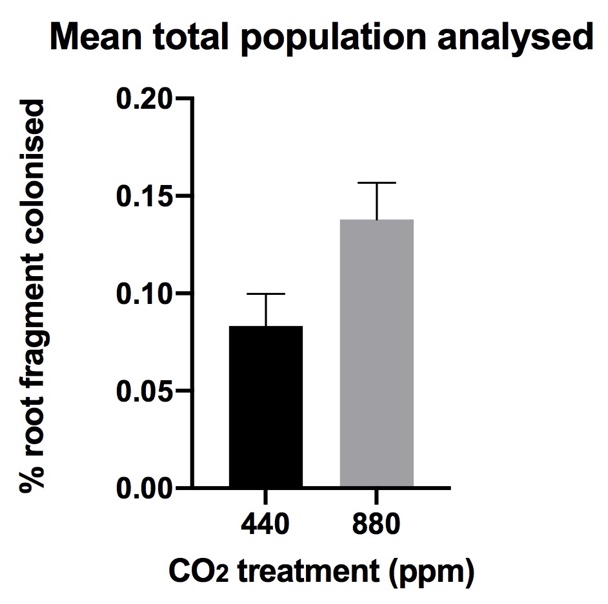


*

c)

**Figure S2**. Comparison of colonisation of root segments colonised by CO2 treatment. a) Proportion of total root segments analysed (Fisher’s exact test, P value <0.01); b) Mean percentage colonised per root fragment (440 = 8.328%; 880 = 13.8%); c) Difference between the population means (0.05469 ± 0.02507; P value < 0.05).
